# A new case of envenomation by neotropical opisthoglyphous snake *Philodryas olfersii* (Lichtenstein, 1823) in Recife, State of Pernambuco, Brazil

**DOI:** 10.1590/0037-8682-0151-2020

**Published:** 2020-06-22

**Authors:** Vanessa do Nascimento Barbosa, Jéssica Monique da Silva Amaral, Álvaro Amon Aquino Alves, Frederico Gustavo Rodrigues França

**Affiliations:** 1 Universidade Federal da Paraíba, Programa de Pós-Graduação em Ecologia e Monitoramento Ambiental, Rio Tinto, PB, Brasil.; 2 Colaborador do Serpentário Mata Sul, Rio Formoso, PE, Brasil.; 3 Universidade Federal da Paraíba, Departamento de Engenharia e Meio Ambiente, Centro de Ciências Aplicadas e Educação, Rio Tinto, PB, Brasil.

**Keywords:** Snakebite, Ophidian accident, Opisthoglyphous

## Abstract

Human envenomation by the snakes Colubridae and Dipsadidae are reported in Brazil, and envenomation by the Opisthoglyphous snake *Philodryas olfersii* could be dangerous. Here, we present the second record of an envenomation by *Philodryas olfersii* in Pernambuco, northeast Brazil. The male victim presented with mild erythema pain, paraesthesia, local numbness, and swollen lymph nodes. The symptoms were similar to those of a pit viper bite, and disappeared completely after 15 days.

## INTRODUCTION

Cases of human envenomation caused by venomous snakes are relatively common worldwide^1^. Although snakebites mainly involve species of the Viperidae and Elapidae families, there have been reports of envenomation caused by opisthoglyphous species of Colubridae and Dipsadidae, such as *Dispholidus typus* and *Thelotornis kirtlandii* in Africa^2^.

In Brazil, approximately 32,000 cases of snakebite were reported in 2019, of which the majority (73%) was caused by venomous snakes (Viperidae), 6% were caused by species considered to be opistoglyphous (Colubridae and Dipsadidae) and 5% by unidentified species^3^. Certain Brazilian opistoglyphous snakes reported as involving human poisoning include *Erythrolamprus* ^4^, *Thamnodynastes* ^5^ and *Philodryas* ^6^.

*Philodryas olfersii* toxin comes from the Duvernoy seromucous gland that facilitates prey immobilization, regardless of the use of constriction^7^. Its venom has a biological activity similar to that of of viper snakes from the genus *Bothrops,* with less intense local action^8^. Instances of envenomation by opistoglyphous snakes have increased considerably over the years^9^ and, consequently, studies have been conducted regarding the biochemical and pharmacological properties of their venom^8^. However, little is known concerning human reactions to envenomation, or the clinical manifestations caused by opistoglyphous dipsadids. In this report, we describe a second envenomation after snakebite by *Philodryas olfersii* of an amateur herpetologist in Recife, State of Pernambuco, northeast Brazil. This poisoning event happened eight years after the first report in that city^6^.

## CASE REPORT

**FIGURE 1:**
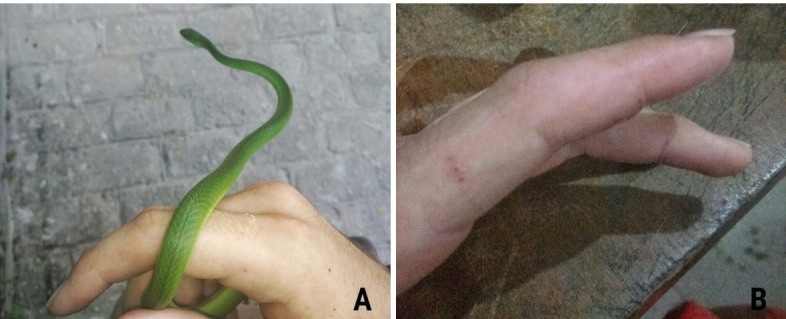
**(A)** The man handling the snake by the middle of its body without a contention; **(B)** Snake bite on index finger of left hand.

On November 1st, 2017 an ophidic envenomation occurred during the handling of a *Philodryas olfersii* snake by a male animal hobbyist (29 years old, 85 kg, 1.77 m) in the Recife municipality, Pernambuco State, northeast Brazil. The snake was slowly moving across the grass in the Parque Estadual de Dois Irmãos. The man took the snake by the middle of its body without a contention (Figure 1A), believing it to be an aglyph - the harmless *Erythrolamprus viridis*. At 15:30 h the snake bit first on the man’s left hand between the pinky and the ring finger, and after five minutes it made a second strike on the index finger (middle phalanx) of the left hand (Figure 1B). In this second bite, the snake ‘chewed’ for approximately five seconds, immediately causing bleeding at the injury sites. After twenty minutes, the patient reported local itching and mild pain involving the lesion, but bleeding stopped. At 15:51 h the pain decreased, but erythema and paresthesia extended to the distal phalanx. Local pain ceased at 16:00, with flushing, increasing paresthesia, and initiation of edema in the proximal, medial and distal phalanges of the index finger. The patient did not seek medical help and returned home. No medication was used during the period of intoxication. After four hours, there was a darkening of the skin near the tip of the finger that lasted for seven days. The finger remained dormant for a period of 12 hours after the envenomation. On November 2^nd^, after 24 h, the patient reported swelling of lymph nodes in the neck that remained for 15 days. Edema in the phalanges disappeared after 48 h. 

## DISCUSSION

Symptoms similar to those reported in the present study, such as pain, edema, bleeding at the injury site, erythema, and paresthesia, from bites of non-venomous opistoglyph snakes, can be confused with symptoms presenting in after pit viper (*Bothrops* spp.) bites, and lead to sometimes inopportune use of anti-ophidic serum^4^. In addition, some pit viper bites can cause lesions that often result in secondary infections by microorganisms that are often associated with the buccal flora of snakes^10^. However, no signs of secondary infections caused by the bite of *P. olfersii* were observed in the present case. Relevant secondary infections caused by non-venomous snakes (Colubridae and Dipsadidae) are less likely to occur because the venom of these animals has no proteolytic action^11^.

**FIGURE 2:**
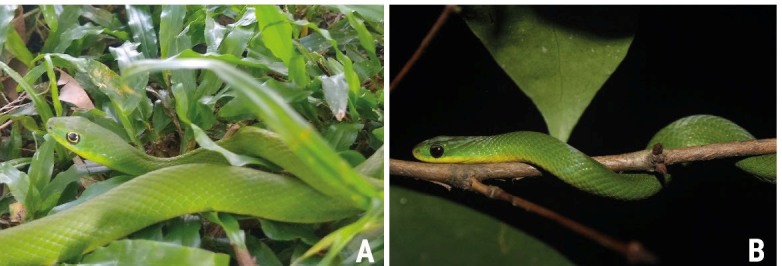
Green colored pattern of *Philodryas olfersii* that occurs in northeastern Brazil, resembling *Erythrolamprus viridis.* (**A)** *P. olfersii;* **(B)** *E. viridis.*

The snake *Philodryas olfersii* (Figure 2A) is common in Brazil, with large populations in the northeast. The snake is diurnal, eats mainly small mammals and lizards, and can be found near houses, close to forests, or near anthropic vegetation^6^, which facilitates encounters with humans. In addition, while the snake has brown spots on the top of the head and a gold vertebral line in populations in southeast and central Brazil, the species is entirely green in the northeast, resembling the harmless snake *Erythrolamprus viridis* (Figure 2B). 

Therefore, our report reinforces the precautions and care that professional or amateur herpetologists must take in handling many opsisthoglyphous snakes. Although the number of envenomations caused by opsisthoglyphous snakes in Brazil is low, and with reduced capacity of injecting venom, some cases of envenomation by opisthoglyphous snakes, such as *Philodryas* species, could be very dangerous and should be avoided. 
